# Prevalence of scoliosis among school students in a town in southern Brazil

**DOI:** 10.1590/S1516-31802010000200005

**Published:** 2010-03-04

**Authors:** Lenice Sberse Nery, Ricardo Halpern, Paulo César Nery, Karin Passos Nehme, Aírton Tetelbom Stein

**Affiliations:** I MSc. Nurse. Specialist Public Health teacher, Campanha Nacional de Escolas da Comunidade (CNEC) and municipal employee in Bento Gonçalves, Rio Grande do Sul, Brazil.; II MD, PhD. Professor of Public Health, Universidade Luterana do Brasil (Ulbra) and Universidade Federal of Ciências da Saúde de Porto Alegre (UFCSPA), Porto Alegre, Rio Grande do Sul, Brazil.; III MD. Orthopedist in Carlos Barbosa, Rio Grande do Sul, Brazil.; IV Physiotherapist in Carlos Barbosa, Rio Grande do Sul, Brazil.; V MD, PhD. Professor of Public Health, Universidade Luterana do Brasil (Ulbra) and Universidade Federal em Ciências da Saúde de Porto Alegre (UFCSPA); care protocol coordinator of Grupo Hospitalar Conceição (GHC), Porto Alegre, Rio Grande do Sul, Brazil.

**Keywords:** Scoliosis, Spine, Primary prevention, Students, Triage, Escoliose, Coluna vertebral, Prevenção primária, Estudantes, Triagem

## Abstract

**Context And Objective:**

Scoliosis is not a diagnosis, but a description of a structural alteration that occurs in a variety of conditions. Progression of the curvature during periods of rapid growth may result in severe deformity, which may be accompanied by cardiopulmonary compromise. This study had the aims of measuring the prevalence of scoliosis among students in the fifth to eighth school years and investigating possible associations between the presence of scoliosis, body overweight and the weight of school materials.

**Design And Setting:**

Analytical cross-sectional study developed in the municipality of Carlos Barbosa, Rio Grande do Sul, Brazil.

**Methods:**

A total of 1340 students were evaluated. The variables studied were the prevalence of scoliosis, type of school, location of the school, age, school year, sex, body weight, prevalence of excessive school material weight, height, body mass index (BMI) and spinal alignment measurements.

**Results:**

The prevalence of scoliosis was 1.4%; shoulder and scapula asymmetry, 6.6%; forearm and trunk asymmetry, 4.0%; spinal misalignment, 1.9%; Thales triangle asymmetry, 6.4%; body overweight, 19.8%; and carrying of excessively heavy school materials, 27%. The study did not find any statistically significant association between scoliosis and body overweight, or between scoliosis and excessive weight of school materials.

**Conclusions:**

The prevalence of scoliosis in this school-based sample was low. No correlation was found between this clinical condition and the other variables.

## RESUMO

**Contexto E Objetivo:**

A escoliose não é um diagnóstico, e sim uma descrição de uma alteração estrutural que ocorre numa variedade de condições. Progressão da curvatura durante o período de rápido crescimento pode resultar numa deformidade grave, a qual pode ser acompanhada por comprometimento cardiopulmonar. O objetivo do estudo foi medir a prevalência de escoliose entre escolares da quinta a oitava série e verificar a possível associação entre a presença de escoliose, o sobrepeso corporal e o peso do material escolar.

**Tipo De Estudo E Local:**

Estudo transversal analítico realizado no município de Carlos Barbosa, Rio Grande do Sul, Brasil.

**MÉtodos:**

Foram avaliados 1.340 escolares. As variáveis estudadas foram a prevalência de escoliose, tipo de escola, local da escola, idade, série, sexo, peso corporal, prevalência de peso excessivo do material escolar, altura, índice de massa corporal (IMC) e medidas de alinhamento da coluna vertebral.

**Resultados:**

A prevalência de escoliose foi de 1,4%; de assimetria de ombros e escápula, 6,6%; de assimetria antebraço e tronco, 4,0%; desalinhamento da coluna vertebral, 1,9%; assimetria do triângulo de Tales, 6,4%; o sobrepeso corporal, 19,8%; e carregar material escolar com peso excessivo, 27%. O estudo não encontrou associação estatisticamente significativa entre a escoliose e o sobrepeso corporal e a escoliose e o excesso de peso do material escolar.

**ConclusÕes:**

A prevalência de escoliose com base escolar mostrou-se baixa. Não foi encontrada correlação entre essa condição clínica e as outras variáveis.

## INTRODUCTION

Scoliosis is a public health problem with a prevalence of 0.5% to 3% among the school population.^1^ It has the capacity to create initial asymptomatic deformities that may remain unnoticed by parents and teachers and it has an undesirable prognosis.

Postural problems relating to the spine originate during childhood and adolescence through physical development.^2,3^ Moreover, during these periods, individuals are likely to present risky behavior in relation to the spine.^4,5^ Back asymmetry often occurs in the adolescent population because of body posture abnormalities. In turn, such abnormalities can be explained by the fact that many body postures in daily use are inadequate for the anatomical structures, thereby increasing the total stress on body elements, and especially on the spine.^6,7^

One common occurrence among adolescents is trunk asymmetry, which can be considered to be the clinical expression of scoliosis. Adolescents and children only seek care when the deformity is visible and therefore represents a serious problem. For this reason, many studies have investigated risk factors for the spine.^3^

Studies carried out in Sweden, the United States, Italy and Brazil have indicated that backpacks cause the body’s center of gravity to shift backwards. The heavier the bag is, the greater the shift will be. The imbalance thus caused is compensated by bending the body forward, which can cause spinal deviation.^8^

Several studies have shown that children and adolescents carry large quantities of school materials in their backpacks.^9-12^ The greatest concern in this respect relates to the consequences that this daily routine can have for musculoskeletal structures, over the medium and long term, given that these individuals are in the midst of skeletal development.

Geographical differences give rise to several scoliosis rates among school students.^13^ Screening is a presupposition within the primary care model. Therefore, low-cost and high-sensitivity procedures directed towards conditions for which there are early and effective interventions, need to be adopted as practices in the healthcare system. From the point of view of primary healthcare, postural assessments provide an opportunity to correct inadequate behavior, thereby making it possible to minimize the consequences from the deformity, while the treatment is underway.

## OBJECTIVE

The present study had the aims of measuring the prevalence of scoliosis among students in the fifth to eighth school years in a town in southern Brazil and investigating possible associations between the presence of scoliosis, body overweight and the weight of school materials.

## MATERIALS AND METHODS

This was a school-based analytical cross-sectional study carried out among a population of 1340 students of both sexes, who were in the fifth, sixth, seventh and eighth school years in schools in the municipality of Carlos Barbosa, Rio Grande do Sul. The total school student population in the year 2008, as estimated by the Municipal Education Department of Carlos Barbosa, was 1402. This population was divided into 12 schools: nine within the urban area and three in the rural area. There were 11 public schools and one private school. This study was carried out in February, March, April and May 2008.

Scoliosis was measured using the Adams test,^14^ which consisted of positioning the student orthostatically, looking forwards in an inspection position, and then asking the student to lean forward with the upper limbs in a relaxed position and the palms of the hands facing each other. The test was considered positive when there was a difference between the right and left rib cages, with the presence of gibbosity.^14,15^ A physical examination on the trunk was carried out to investigate asymmetries, by viewing the midline of the body (the spine) and comparing the right and left sides.

All the school materials carried by the school students, which consisted basically of books, notebooks and pencil cases, were weighed on the assessment day. The weight was considered excessive if it exceeded 10% of the students’ own weight.^10,11^ In addition, other variables such as weight, height, body mass index (BMI), sex, age, school year, type of school and school location were investigated.

To gather data, a structured questionnaire asking about socioeconomic, demographic, anthropometric and behavioral factors was used, along with physical examination of the posterior part of the trunk (Appendix A). The students’ body mass index (BMI) was measured and stratified by age as recommended by the Ministry of Health. Overweight was defined according to the BMI cutoff points for sex and age. To measure weights and heights, a Walmy digital scale of precision 100 g was used. The same scale was used to weigh the school materials carried by the students. The examination was carried out by trained interviewers (the authors: nurses, MD and physiotherapist). The reliability among the examiners was measured in order to ensure the quality of the examinations, and a kappa coefficient greater than 0.9 was obtained.

The data were stored in a database in the EpiData^16^ software. The SPSS/PC 10.0 software was used for the statistical analysis. To describe the distribution of the variables, central trend measurements were used, such as the mean, median, mode and minimum and maximum of the amplitude range. To investigate associations between asymmetries, the weight of school materials and scoliosis, the chi-square test was used. The significance level was taken to be p < 0.05. The sample size was large enough to ensure an explanation power of at least 90% with an alpha error of about 2%.

This research project was assessed by the Research Ethics Committee of Universidade Luterana do Brasil (Ulbra) and was approved under the protocol number 2008/035H. The aim of the study, its procedures and the voluntary nature of participation were duly informed to the parents and/or other adults responsible for the school students, by means of a free and informed consent statement that was in accordance with National Health Board Resolution number Conselho Nacional de Saúde (CNS) 196/1996. The parents or other adults holding this responsibility signaled their acceptance by signing the statement. Only the students who came to school on the day set for the assessment with the consent agreement duly signed took part in this study. When scoliosis was detected, the adults responsible for the students were informed and the subjects were referred to the healthcare services available in the area.

## RESULTS

Out of the 1402 students attending the school years considered in this study, 1340 were assessed. Losses and refusals amounted to 4.43% (n = 62), including school students who missed the assessment, those whose parents or other adults responsible for them refused to allow participation and those who said they did not want to participate at the time of the assessment.

The study population included 684 boys (51%), the mean age was 12.7 years and the mean BMI was 19.8 kg. The distribution according to school year was that 29.6% of the school students were in the fifth year, 22.8% were in the sixth year, 26.3% were in the seventh year and 21.3% were in the eighth year.

Regarding the type of school, 4.92% were studying in a private school, 33.88% were in municipal schools and 61.19% were in state schools; 21.49% were in schools in the rural zone of the municipality and 78.51% were in schools in the urban zone, as shown in **Table 1**. The frequency of scoliosis was 1.4% (n = 19), and it was more prevalent among girls, with a frequency of 1.98% (n = 13). The frequency of shoulder and scapula asymmetry was 6.6% (n = 89); forearm and trunk asymmetry, 10.4% (n = 140); spinal asymmetry, 1.9% (n = 25); and scalene muscle asymmetry, 6.4% (n = 86), as shown in **Table 2**.

**Table 1.** Sociodemographic characteristics of fifth to eighth-year school students in the municipality of Carlos Barbosa (2008)

Variable	n	%
Sex		
Male	684	51.0%
Female	656	49.0%
School location		
Urban	1042	77.8%
Rural	298	22.2%
Type of school		
Private	66	4.9%
Municipal	454	34.0%
State	820	61.1%
Grade		
5^th^	396	29.6%
6^th^	306	22.8%
7^th^	352	26.3%
8^th^	286	21.3%

**Table 2.** Frequency of scoliosis and asymmetries among fifth to eighth-year school students in the municipality of Carlos Barbosa (2008)

Asymmetries	n	%
Scoliosis	19	1.4
Shoulder and scapula asymmetry	89	6.6
Spine asymmetry	25	1.9
Forearm and trunk asymmetry	140	10.4
Thales triangle asymmetry	86	6.4

The prevalence of excessive weight of school materials was found to be 27%. i.e. 41%, 29% and 18% in private, municipal and state schools, respectively, as shown in **Table 3**. The study indicated that among the students who did not have scoliosis, the weight of the school materials was 3.7 kg, with a body weight of 48 kg. However, among the students with scoliosis, the weight of the school materials was 4.4 kg, with a body weight of 47.9 kg.

**Table 3.** Student distribution according to excessive weight of school materials, in relation to type of school, in the municipality of Carlos Barbosa (2008)

	Private	Municipal	State
Excess	28	239	82
No excess	38	581	372
**Total**	**66**	**820**	**454**

The study found a marginal statistical association between presence of scoliosis and the weight of the school materials (P = 0.08), as shown in **Table 4**. In this association, the students carrying excessive weight in their backpacks presented an estimated risk (prevalence rate, PR) of 2.13, with a 95% confidence interval (CI) of 0.8-5.2, compared with students who did not carry excessive weight.

**Table 4.** Sample distribution according to asymmetries, in relation to excessive weight of school materials and body overweight, in the municipality of Carlos Barbosa (2008)

Asymmetries		Excessive weight of school materials							Body overweight						
		yes		no		total		P	yes		no		total		P
		n	%	n	%	n	%		n	%	n	%	n	%	
Scoliosis	yes	8	42	11	58	19	100	0.08	3	16	16	84	19	100	0.50
	no	333	25	987	75	1320	100		254	19	1066	81	1320	100	
Shoulder and scapula	yes	23	25	66	75	89	100	0.50	22	25	67	75	89	100	0.20
	no	318	25	932	75	1250	100		236	19	1014	100	1250	100	
Forearm and trunk	yes	5	9.2	49	90.8	336	26	0.02	10	18.5	44	81.5	54	100	1.00
	no	336	26	949	74	1003	100		247	19	1038	81	1285	100	
Thales triangle	yes	20	15	73	75	86	100	1.00	28	33	58	67	86	100	0.00
	no	328	27	918	73	1253	100		229	18	1024	82	1253	100	

The prevalence of shoulder and scapula asymmetry and excessive weight of school materials was found to be 44% in the private school, 5.0% in the municipal schools and 4.0% in the state schools, as can be seen in **Table 5**.

**Table 5.** Student distribution according to shoulder and scapula asymmetry, in relation to type of school, in the municipality of Carlos Barbosa (2008)

	Private	Municipal	State
Excess	29	39	20
No excess	37	781	434
**Total**	**66**	**820**	**454**

The frequency of body overweight was 19.8%. When the subjects were assessed regarding the possible association of body overweight with scoliosis, no statistically significant association was found between these two study variables. However, there was a statistically significant association between body overweight and scalene muscle asymmetry (P = 0.00).

## DISCUSSION

The prevalence of scoliosis among school students between 10 and 14 years of age, of both sexes, who were attending the fifth, sixth, seventh and eighth years in schools in the municipality of Carlos Barbosa was 1.4% (n = 19). This prevalence is in accordance with the prevalence found in previous studies, which has ranged from 1% to 13% worldwide among the general population.^17^ Among school populations, the prevalence has ranged from 0.5% to 3%.^1^

The present study found similarities with the results from a study in São Paulo that examined 4,037 students using the Adams method. The teachers mentioned a prevalence of 19.8%, the physician mentioned 2.4% and the radiographic examination showed a prevalence of 1.8%.^18^

Regarding the differences in the prevalence of scoliosis, it is possible that behavioral factors are strongly associated with this deformity. A study in Sobral (Ceará, Brazil) stated that children and adolescents were in the habit of sleeping in hammocks,^24^ while a study in southern Brazil found associations between scoliosis and the number of hours on the computer and between scoliosis and body overweight.^8^ Another study in Santa Catarina, Brazil, associated the problem with backpack overweight and with inadequate school furniture.^8,9^

Another factor that may have an influence on establishing scoliosis is the method used to identify it. There would be a possibility of finding a higher prevalence of scoliosis using the Adams test than using radiographic examinations. It is possible that the inter-examiner assessment method provides a more precise measurement for identifying the deformity. A second assessment to confirm the deformity may also help in obtaining the prevalence measurement.

Another study had findings similar to those of the present study. Soucacos et al.,^19^ in Greece, analyzed students between 9 and 15 years of age and found a prevalence of 1.7%. Andrade Barcia and Andrade Barcia^20^ carried out a study on primary and secondary school students in Ecuador and found a prevalence of 0.88%, i.e. smaller than in the present study.

The prevalence of scoliosis in countries such as Argentina^21^ and Greece^19^ was less than 2%, whereas in Brazil, Rocha and Pedreira observed a prevalence of 7.32%.^22^ Vernengo Lezica^21^ carried out a study on students between 10 and 15 years of age in Buenos Aires, Argentina. Over a three-year period, this author assessed 9,429 students in 140 schools and found that 126 of them had scoliosis (2.6%).

In other similar studies, Ferriani et al.^23^ found a prevalence of scoliosis of 23.5% using the Adams test in Ribeirão Preto, São Paulo, whereas Tavares et al.^24^ found a prevalence of scoliosis of 4.8% in Sobral, Ceará.

The present study showed a prevalence of 1.98% (n = 13) among girls and 0.8% among boys, with proportions of 2.4 per 1. It is possible this difference occurred because the peak body growth among females occurs during the age range studied, thus making it easier to view the deformity. Among males, the growth peak occurs later on.

In a study at Pedro Ernesto University Hospital, Universidade do Estado do Rio de Janeiro (UERJ), with the aim of diagnosing early scoliosis among adolescents, Elias and Texeira^25^ assessed a total of 4,750 adolescents. Among the 85 individuals (1.78%) who presented positive clinical signs, 54 underwent a radiological assessment and the diagnosis was confirmed in the cases of 49 of them (1.03%). The female sex was the most affected, in proportions of 2:1.

No statistically significant association between scoliosis and excessive weight of school materials was found, and one possible explanation for the marginal association may have been the low prevalence of scoliosis. This differed from the findings of Alvarez Núñez and Oquendo Vázquez,^26^ who carried out a study among 2,000 school students in Matanzas, Cuba, to investigate the incidence of scoliosis and analyze the school-based factors that might give rise to risks to the spine, and observed that scoliosis was present in 10.4% of the students. They stated that the weight of school materials was a risk factor.

In spite of the marginal significance that we obtained for the relationship between the excessive weight carried by the school students and shoulder and scapula asymmetries, we noticed that in the private school, where the weight of school materials was greater, the frequency of this asymmetry was also greater, which may be a contributory factor for the presence of scoliosis curves.

In the same way, the excessive weight of school materials presented a statistically significant association with forearm and trunk asymmetry (P = 0.02), thus suggesting that excessive weight carried may be connected with asymmetry. Therefore, the forearm and trunk asymmetry and the scalene muscle asymmetry (P = 0.00), which presented a statistically significant association with scoliosis, showed that such asymmetries may be a source of concern, since they may indicate future problems.

The present study presents limitations inherent to cross-sectional studies. The school materials were only weighed on the assessment day, for reasons of operational convenience. A weekly average might be more accurate with regard to the students’ habits, thereby minimizing the possible effect of dispersion of the weight of school materials over the weekly curricular schedule. In addition, no radiographic examinations of a more specific confirmatory nature were carried out.

## CONCLUSION

This study demonstrated that body overweight may be associated with shoulder and scapula asymmetries and with scalene muscle asymmetries and suggests that there is a need for preventive action regarding body weight care and control. Excessive weight carried by school students seems to have an association with forearm and trunk asymmetry and suggests that there is a need for intervention to maintain trunk balance.

Therefore, routine trunk assessment actions in schools are very important, because they can lead to intervention in the process of abnormality formation, through early corrections, since such asymmetries are easy for examiners to see and are noninvasive. The time taken to perform the examination is short and the cost is low. Health and education professionals need to be involved in the scoliosis screening process for students and the guidance for reducing the risk needs to be emphasized.

## Appendix A. Questionnaire to gather data on the study variables

**Table:**

Name:					
School:					Phone number:
1. Student’s date of birth					DB
2. Sex	1. male	2. female			SEX
3. Body weight _________ kg					BW
4. School year	5^th^	6^th^			G
	7^th^	8^th^			
5. School location	1. urban				
	2. rural				SLOC
6. Type of school	1. Public				
	2. Private				KS
7. Height					H
8. Body mass index					BMI
9. Weight of school materials			kg		WSS
10. Shoulder and scapula symmetry			1. Yes	2. No	SBS
11. Forearm and trunk symmetry			1. Yes	2. No	FTS
12. Spinal alignment			1. Yes	2. No	SA
13. Thales triangle symmetry			1. Yes	2. No	SMS
14. Presence of gibbosity			1. Yes	2. No	PG
15. Presence of scoliosis			1. Yes	2. No	PS

## References

[1] Rocha LE, Santili C, Carrera EF (2004). Escolioses idiopáticas. In: Sociedade Brasileira de Ortopedia e Traumatologia. Ortopedia pediátrica.

[2] Bunnell WP (2005). Selective screening for scoliosis. Clin Orthop Relat Res.

[3] Nissinen MJ, Heliövaara MM, Seitsamo JT, Könönen MH, Hurmerinta KA, Poussa MS (2000). Development of trunk asymmetry in a cohort of children ages 11 to 22 years. Spine (Phila Pa 1976).

[4] Shehab DK, Al-Jarallah KF (2005). Nonspecific low-back pain in Kuwaiti children and adolescents: associated factors. J Adolesc Health.

[5] Detsch C, Luz AMH, Candotti CT (2007). Prevalência de alterações posturais em escolares do ensino médio em uma cidade no Sul do Brasil [Prevalence of postural changes in high school students in a city in southern Brazil]. Rev Panam Salud Pública = Pan Am J Public Health.

[6] Hakala P, Rimpelä A, Salminen JJ, Virtanen SM, Rimpelä M (2002). Back, neck, and shoulder pain in Finnish adolescents: national cross sectional surveys. BMJ.

[7] Gross J, Fetto J, Rosen E (2000). Exame musculoesquelético.

[8] Perez V (2002). A influência do mobiliário e da mochila escolares nos distúrbios músculo-esqueléticos em crianças e adolescentes. [dissertation]. http://www.educacaofisica.com.br/download.asp?tp=biblioteca&id=1737.

[9] Rebelatto JR, Caldas MAJ, Vitta A (1991). Influência do transporte do material escolar sobre a ocorrência de desvios posturais em estudantes [Influence of schoolar material transport on postural deviations occurrence in students]. Rev Bras Ortop.

[10] De Vitta A, Madrigal C, Sales VS (2003). Peso corporal e peso do material escolar transportado por crianças em idade escolar [Corporal weight and school material weight transported by children in school age]. Fisioter Mov.

[11] Negrini S, Carabalona R, Sibilla P (1999). Backpack as a daily load for schoolchildren. Lancet.

[12] Negrini S, Carabalona R (2002). Backpacks on! Schoolchildren’s perceptions of load, associations with back pain and factors determining the load. Spine (Phila Pa 1976).

[13] World Health Organization The World Health Report, 2002. Reducing risks, promoting healthy life. http://epsl.asu.edu/ceru/Documents/whr_overview_eng.pdf.

[14] Adams W (1865). Lectures on the pathology and treatment of lateral and other forms of curvature of the spine.

[15] Ferreira R (2008). Avaliação ortopédica para a coluna vertebral em crianças. Sociedade Brasileira de Ortopedia e Traumatologia.

[16] Lauritsen JM, Bruus M EpiData Entry (version 3). A comprehensive tool for validated entry and documentation of data. The Epidata Associaton.

[17] Brooks HL, Azen SP, Gerberg E, Brooks R, Chan L (1975). Scoliosis: A prospective epidemiological study. J Bone Joint Surg Am.

[18] Martini S, Ortiz J (1993). Avaliação escolar de escoliose: uso de cartaz educativo [School evaluation of scoliosis: use of educational poster]. Rev Bras Ortop.

[19] Soucacos PN, Soucacos PK, Zacharis KC, Beris AE, Xenakis TA (1997). School-screening for scoliosis. A prospective epidemiological study in northwestern and central Greece. J Bone Joint Surg Am.

[20] Andrade Barcia A, Andrade Barcia J (1996). Escoliosis estructural idiopática y otras alteraciones esqueléticas en escolares de los niveles primario y secundario de la ciudad de portoviejo. Educ Méd Contin.

[21] Vernengo Lezica A (1994). Detección precoz de deformidades de columna en escolares de 10 a 15 años. Rev Asoc Argent Ortop Traumatol.

[22] Rocha EST, Pedreira ACS (2001). Problemas ortopédicos comuns na adolescência [Common orthopedic problems in adolescence]. J Pediatr (Rio J).

[23] Ferriani MGC, Cano MAT, Candido GT, Kanchina AS (2000). Levantamento epidemiológico dos escolares portadores de escoliose da rede pública de ensino de 1° grau no município de Ribeirão Preto. Revista Eletrônica de Enfermagem.

[24] Tavares ARA, Feitosa EL, Bezerra LMM (2001). Proposta de implantação do fisioterapeuta na escola face a alterações posturais. Revista Coluna Fisioterápica.

[25] Elias N, Teixeira JCM (1992). Escoliose idiopática do adolescente: diagnóstico precoce através do exame ortopédico rotineiro [Idiopathic scoliosis of the adolescent: early diagnosis by routine orthopedic examination]. Rev Bras Ortop.

[26] Alvarez Núñez R, Oquendo Vázquez P (1988). Factores escolares predisponentes en la escoliosis idiopática [Predisposing school factors in idiopathic scoliosis]. Rev Cuba Pediatr.

